# Comprehensive analysis of microRNA-regulated protein interaction network reveals the tumor suppressive role of microRNA-149 in human hepatocellular carcinoma via targeting AKT-mTOR pathway

**DOI:** 10.1186/1476-4598-13-253

**Published:** 2014-11-26

**Authors:** Yanqiong Zhang, Xiaodong Guo, Lu Xiong, Lingxiang Yu, Zhiwei Li, Qiuyan Guo, Zhiyan Li, Boan Li, Na Lin

**Affiliations:** Institute of Chinese Materia Medica, China Academy of Chinese Medical Sciences, Beijing, 100700 China; 302 Hospital of PLA, Beijing, 100039 China; Beijing Zhongguancun Hospital, Beijing, 100190 China

**Keywords:** Hepatocellular carcinoma, microRNA-regulated protein interaction network, microRNA-149, AKT/mTOR pathway, Prognosis

## Abstract

**Background:**

Our previous study identified AKT1, AKT2 and AKT3 as unfavorable prognostic factors for patients with hepatocellular carcinoma (HCC). However, limited data are available on their exact mechanisms in HCC. Since microRNAs (miRNAs) are implicated in various human cancers including HCC, we aimed to screen miRNAs targeting AKTs and investigate their underlying mechanisms in HCC by integrating bioinformatics prediction, network analysis, functional assay and clinical validation.

**Methods:**

Five online programs of miRNA target prediction and RNAhybrid which calculate the minimum free energy (MFE) of the duplex miRNA:mRNA were used to screen optimized miRNA-AKT interactions. Then, miRNA-regulated protein interaction network was constructed and 5 topological features (‘Degree’, ‘Node-betweenness’, ‘Edge-betweenness’, ‘Closeness’ and ‘Modularity’) were analyzed to link candidate miRNA-AKT interactions to oncogenesis and cancer hallmarks. Further systematic experiments were performed to validate the prediction results.

**Results:**

Six optimized miRNA-AKT interactions (miR-149-AKT1, miR-302d-AKT1, miR-184-AKT2, miR-708-AKT2, miR-122-AKT3 and miR-124-AKT3) were obtained by combining the miRNA target prediction and MFE calculation. Then, 103 validated targets for the 6 candidate miRNAs were collected from miRTarBase. According to the enrichment analysis on GO items and KEGG pathways, these validated targets were significantly enriched in many known oncogenic pathways for HCC. In addition, miRNA-regulated protein interaction network were divided into 5 functional modules. Importantly, AKT1 and its interaction with mTOR respectively had the highest node-betweenness and edge-betweenness, implying their bottleneck roles in the network. Further experiments confirmed that miRNA-149 directly targeted AKT1 in HCC by a miRNA luciferase reporter approach. Then, re-expression of miR-149 significantly inhibited HCC cell proliferation and tumorigenicity by regulating AKT1/mTOR pathway. Notably, miR-149 down-regulation in clinical HCC tissues was correlated with tumor aggressiveness and poor prognosis of patients.

**Conclusion:**

This comprehensive analysis identified a list of miRNAs targeting AKTs and revealed their critical roles in HCC malignant progression. Especially, miR-149 may function as a tumor suppressive miRNA and play an important role in inhibiting the HCC tumorigenesis by modulating the AKT/mTOR pathway. Our clinical evidence also highlight the prognostic potential of miR-149 in HCC. The newly identified miR-149/AKT/mTOR axis might be a promising therapeutic target in the prevention and treatment of HCC.

**Electronic supplementary material:**

The online version of this article (doi:10.1186/1476-4598-13-253) contains supplementary material, which is available to authorized users.

## Background

Hepatocellular carcinoma (HCC) ranks as the fifth (seventh) most prevalent malignancy and the second (sixth) leading cause of cancer-related deaths in men (women), accounting for approximately 695,900 deaths per year worldwide [1]. The hepatocarcinogenesis is a multi-step process from chronic hepatitis, cirrhosis and dysplastic nodules to malignant tumors [2]. Despite the great advancement of numerous therapeutic strategies, HCC remains a major public health concern due to fast infiltrating growth, early metastasis, high-grade malignancy, and poor prognosis. More than two-thirds of HCC patients occur recurrence after surgical hepatic resection [3]. The overall 5-year survival rate for these patients is still only 5% [4]. Growing clinical observations indicate that HCC patients with the same clinicopathologic features often display different outcome, suggesting that there may be several complex molecular and cellular events involved in the development and aggressive progression of HCC. Therefore, it is of crucial significance to investigate the molecular pathogenesis of HCC in order to develop novel therapeutic strategies for the treatment of this disease.

MicroRNAs (miRNAs) represent a group of short non-coding RNA molecules with 18–25 nucleotides in length [5]. Based on miRBase (release 21), the human genome encodes 1881 (2588) precursor (mature) miRNAs, which potentially target the majority of the human genes [6]. Functionally, miRNAs transcriptionally or post-transcriptionally suppress the expression of their target genes at mRNA or protein levels in many organisms, such as yeast, fruit flies, worms, vertebrates, human and plants by imperfect basepairing with the 3′untranslated regions (3′UTRs) of target genes [7], and play important roles in regulating diverse biological processes, including development, cell cycle, proliferation, differentiation, apoptosis and response to stress [8]. In recent years, accumulating evidence have also indicated that the dysregulation of miRNAs in various human cancers may modulate tumor cell proliferation, tumor angiogenesis, invasion and metastasis during tumor initiation and progression [9]. According to the functions of their target genes, miRNAs either act as oncomiRs or as tumor suppressors [10]. Since a growing number of HCC-related genes have been demonstrated to be regulated by miRNAs, it is not surprising that changes in the endogenous expression of miRNAs may be one of the most crucial mechanisms in the hepatocarcinogenesis. For example, miR-105 has been reported to function as a potential tumor suppressor and inhibit cell proliferation by regulating the PI3K/AKT signaling pathway in human HCC [11]; Tan and colleagues identified a serum of miRNA panel (hsa-miR-206, hsa-miR-141-3p, hsa-miR-433-3p, hsa-miR-1228-5p, hsa-miR-199a-5p, hsa-miR-122-5p, hsa-miR-192-5p, and hsa-miR-26a-5p) that has considerable clinical value in HCC diagnosis [12]; The presence of miR-101 has also been indicated to be a biochemical marker for monitoring the progression of tumor development in HBV-related HCC, and to be a potential prognostic marker and therapeutic target for HCC [13]. These previous findings highlight the central and potential roles of miRNAs in the pathogenesis of HCC and elucidate new possibilities that may be useful as diagnostic and prognostic markers, as well as novel therapeutic targets in HCC. However, the existing knowledge of miRNA alterations in human HCC remains limited.

**Figure 1:**
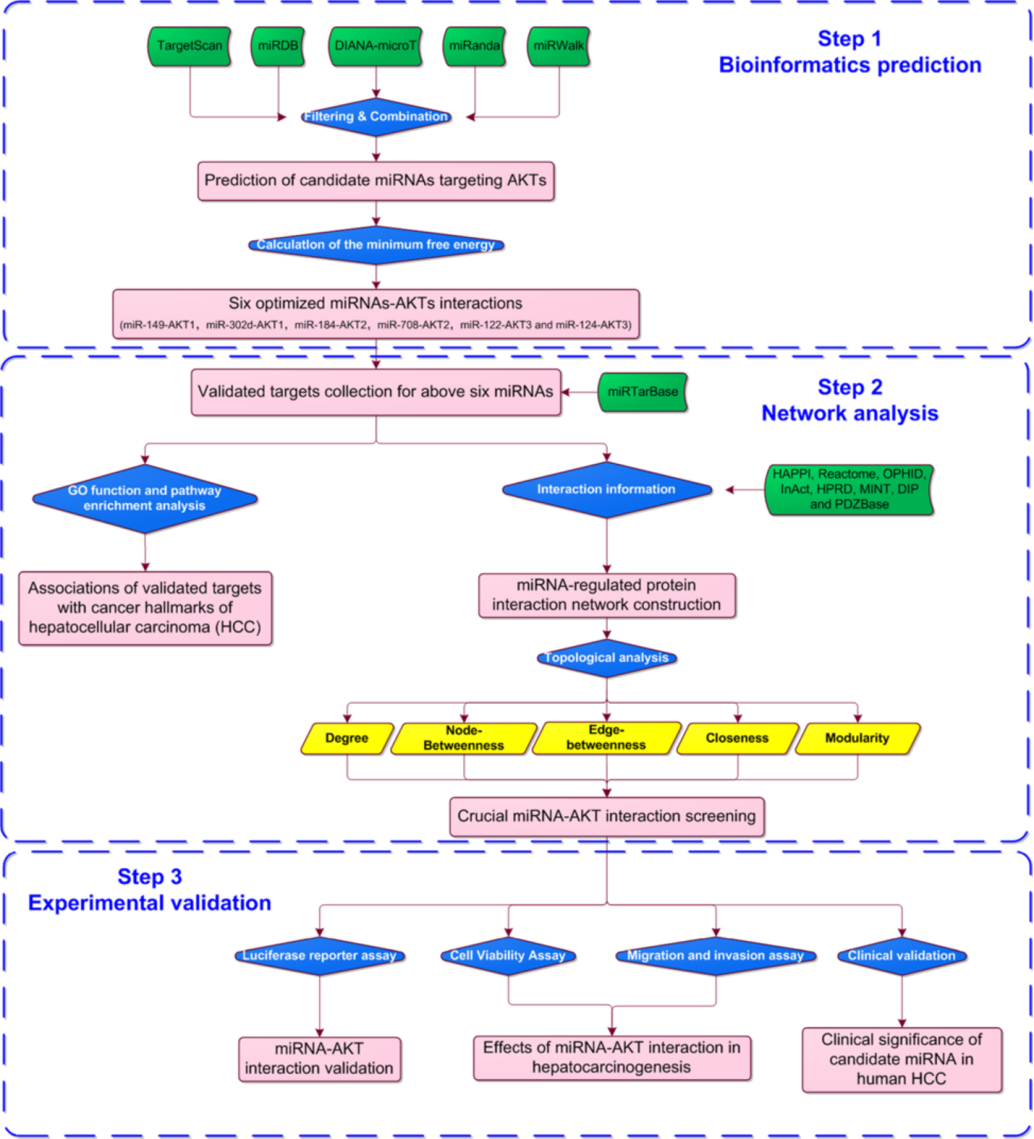
**A schematic diagram of this comprehensive analysis.**

MiRNAs have been demonstrated to function in a multiple-to-multiple relationship with their target genes, which means that a specific miRNA can regulate expression of up to thousand mRNAs, and a specific mRNA can be regulated by multiple miRNAs, implying that the interference of miRNAs in biological processes is quite complicated [14]. Since miRNAs and their target mRNAs function cooperatively, we should construct the miRNAs regulatory networks in order to scrutinize the effects of miRNAs by the network-based systems biology approaches. Our previous study combined expression profile, interaction network analysis and clinical validation to identify three AKT kinase family members (AKT1 ~ AKT3) as unfavorable prognostic factors for HCC patients [15]. However, limited data are available on their exact mechanisms in this cancer. To investigate this issue, we here performed a comprehensive analysis by integrating bioinformatics prediction of miRNAs targeting AKTs, miRNAs-regulated protein interaction network construction and topological analysis, and extensive validation on the miRNA-AKT functional interaction and their clinical relevance in human HCC as shown in Figure 1.

## Results

### Candidate miRNAs targeting AKTs

A total of 29 miRNAs were predicted to respectively target AKT1, AKT2 and AKT3 by more than 3 out of 5 different programs (TargetScan, miRDB, DIANA-microT, miRanda and miRWalk) were listed in Additional file 1: Table S1. Then, RNAhybrid was used to calculate the hybridization free energy of the duplex miRNA:mRNA. The candidate miRNAs were thus confirmed according to the minimum free energy (MFE) values of their interactions with AKTs. As shown in Additional file 2: Table S2, 6 optimized miRNA-AKT interactions (miR-149-AKT1, miR-302d-AKT1, miR-184-AKT2, miR-708-AKT2, miR-122-AKT3 and miR-124-AKT3) had lower MFEs than the median values [-33.35(kcal/mol)] of all MFEs, thus, miR-149 and miR-302d were identified as candidate miRNAs targeting AKT1, miR-184 and miR-708 were identified as candidate miRNAs targeting AKT2, and miR-122 and miR-124 were identified as candidate miRNAs targeting AKT3.

### Enrichment analysis of validated targets for candidate miRNAs

To elucidate the functions of 6 candidate miRNAs, 103 validated targets for them were collected from miRTarBase. Please see detail information on these validated targets in Additional file 3: Table S3. Then, the enrichment analysis based on GO annotation system was performed. This system uses a controlled and hierarchical vocabulary to assign function to genes or gene products in any organism. As shown in Additional file 4: Figure S1A, the validated targets for 6 candidate miRNAs had enriched GO terms related to apoptosis, cell death, and cell cycle. In terms of pathway information which is important for understanding gene and protein function, the validated targets were significantly enriched in many known oncogenic pathways for HCC, such as focal adhesion, cell cycle, p53 signaling pathway, mTOR signaling pathway, apoptosis, VEGF signaling pathway, etc. (Additional file 4: Figure S1B).

### Network analysis

The interaction information of proteins encoded by validated targets of 6 candidate miRNAs were used to construct candidate miRNAs-regulated protein interaction network, which consists of 2868 nodes and 4779 edges. Please see detail information on this network in Additional file 5: Table S4.

**Figure 2:**
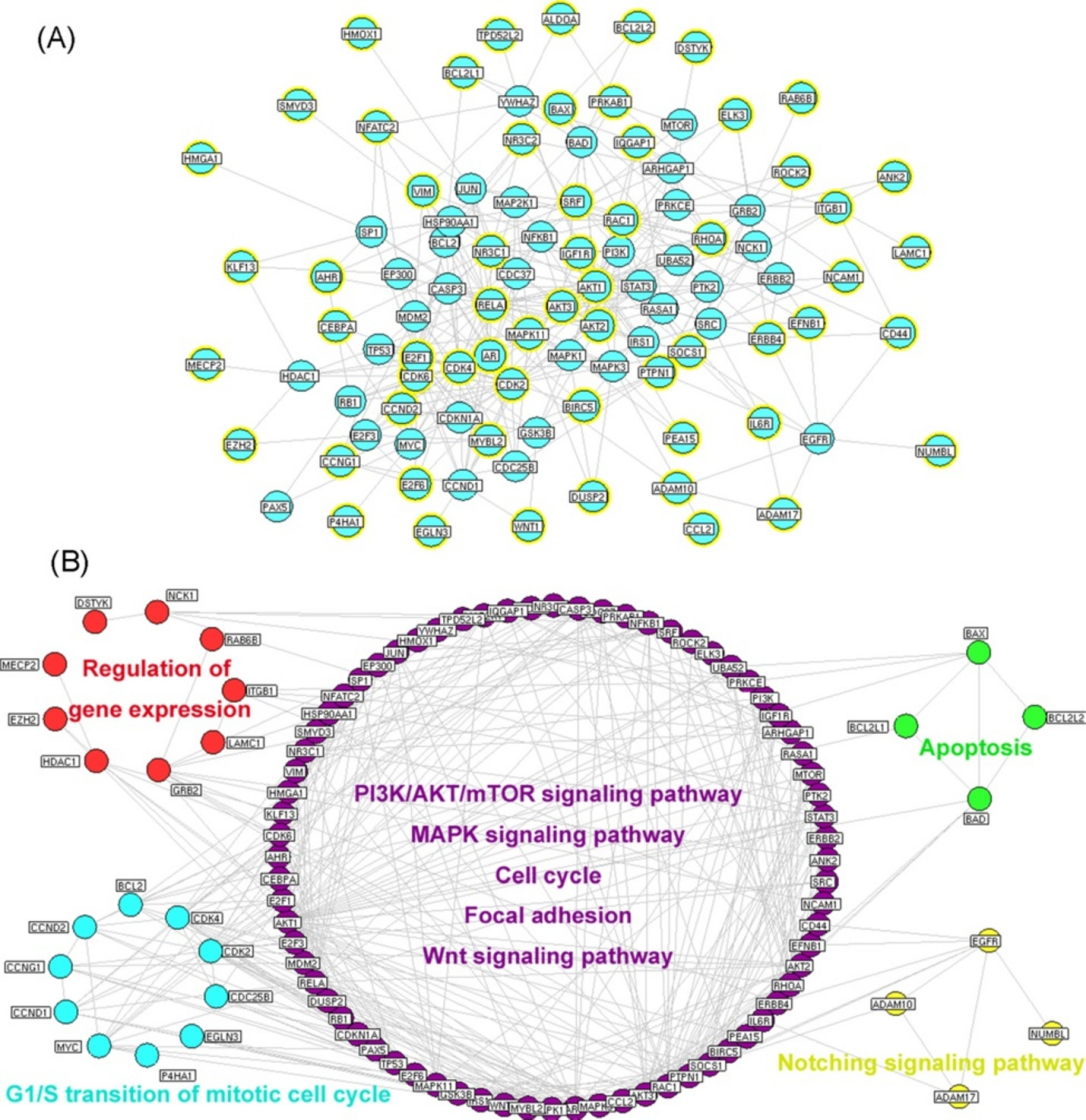
**Candidate miRNAs regulated protein interaction networks. (A)** Interaction network of hub nodes in the candidate miRNAs regulated protein interaction networks. Nodes with yellow rings refer to the validated targets of hsa-miR-149, hsa-miR-302d, hsa-miR-184, hsa-miR-708, hsa-miR-122 and hsa-miR-124. **(B)** Five functional modules of interaction network of hub nodes in the candidate miRNAs regulated protein interaction networks.

Hub proteins in the protein interaction networks have extremely high levels of degree and tend to encode essential genes. According to the previous studies of Li et al. [16] and our research group [15, 17], we identified a node as a hub protein if its degree is more than 2 fold of the median degree of all nodes in a network. As a result, 113 nodes were identified as hub proteins. Then, we constructed the interaction network of these hub proteins, which consists of 99 nodes and 355 edges as shown in Figure 2A. Please see detail information on this network in Additional file 6: Table S5.

Three topological features, ‘Degree’, ‘Node betweenness’ and ‘Closeness’ (defined in ‘Materials and methods’ section) were chosen to identify topological important nodes. After calculating the value of the 3 features for each hub node, the median values of ‘Degree’, ‘Node betweenness’ and ‘Closeness’ were 7, 1.07 and 38.13, respectively. Therefore, we determined that hub nodes with ‘Degree’ > 7, ‘Node betweenness’ > 1.07, and ‘Closeness’ > 38.13 were topological important nodes. As a result, 28 topological important nodes were selected (Additional file 7: Table S6). Among them, 16 (AKT1, AKT2, AKT3, AR, CDK2, CDK4, CDK6, E2F1, IGF1R, MYBL2, NR3C1, PTPN1, RAC1, RELA, RHOA and SOCS1) were validated targets for candidate miRNAs targeting AKTs.Modularity has been reported to be another important aspect of a protein interaction network. Nodes that are highly interconnected within the network are usually involved in the same biological modules or pathways. Using Markov clustering algorithm, we divided the interaction network of hub proteins into 5 functional modules containing 72, 10, 9, 4 and 4 nodes, respectively (Figure 2B). According to the enrichment analysis based on Gene Ontology (GO) annotation system and KEGG pathway, the biggest functional module was significantly associated with PI3K/AKT/mTOR signaling pathway, MAPK signaling pathway, cell cycle, focal adhesion and Wnt signaling pathway. The other modules were respectively involved in G1/S transition of mitotic cell cycle, regulation of gene expression, apoptosis and Notching signaling pathway.

Since an interaction with a high ‘edge-betweenness’ has been defined as a bottleneck which has many ‘shortest paths’ going through it and controls the rate of information flow, we further calculated the ‘edge-betweenness’ of each interaction in the interaction network of hub proteins. As shown in Additional file 8: Table S7, the interaction between AKT1-mTOR had the highest edge-betweenness value (164.38), suggesting its importance in connecting different modules in the network.

Taken together, the above network analysis showed that AKT1 and its interaction with mTOR respectively had the highest node-betweenness and edge-betweenness, implying their bottleneck roles in the network. According to the combination of the miRNA target prediction and MFE calculation, miR-149 and miR-302d were identified as candidate miRNAs targeting AKT1. Pan et al. [18] reported the regulatory effect of miR-149 on AKT1 in glioma cells. Lin et al. [19] also identified AKT1 as a direct target of miR-149* in various cancer cells, including neuroblastoma and Hela cells. However, the interaction between miR-149 and AKT1 has not been validated in HCC cells. In addition, the regulatory effect of miR-302d on AKT1 has not been reported previously. On this context, we would like to perform extensive experiments to confirm the miR-149-AKT1-mTOR axis and its clinical relevance in human HCC.

### Experimental validation

#### MiRNA-149 directly targets AKT1 in HCC cells

**Figure 3:**
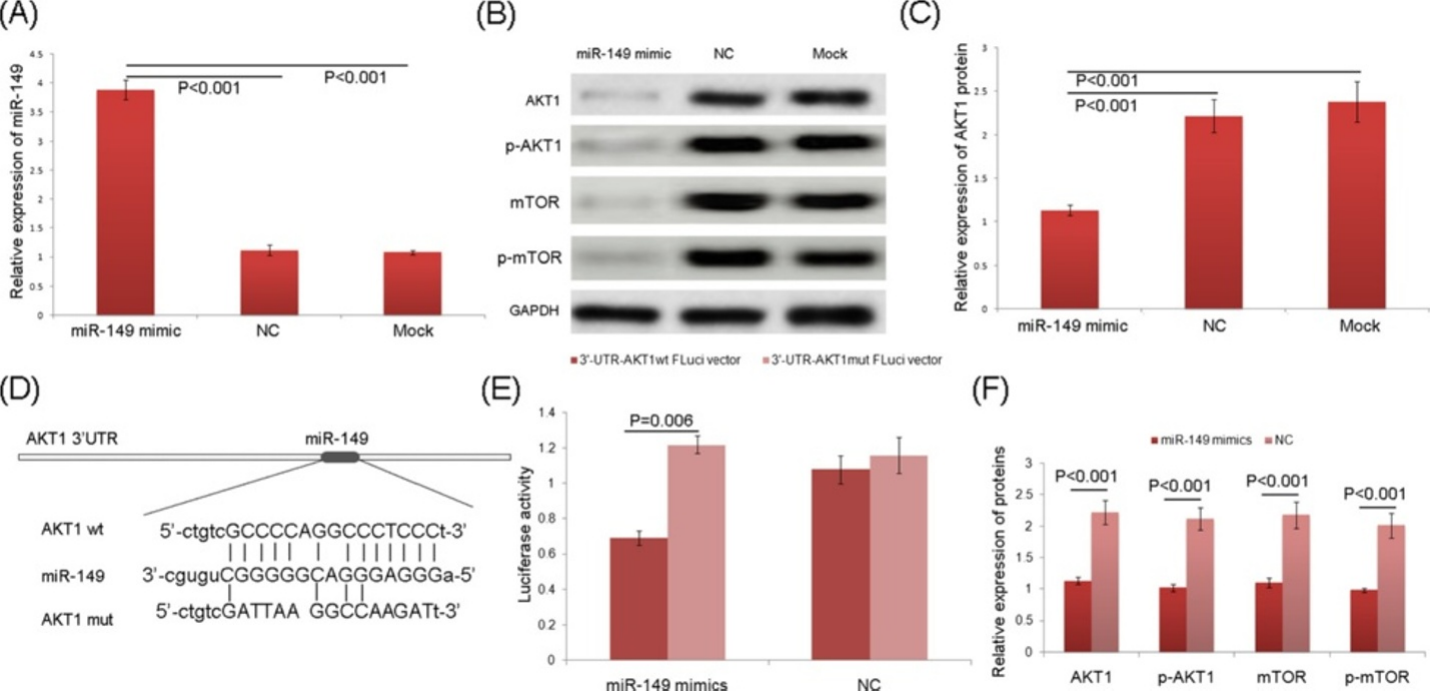
**MiRNA-149 directly targets AKT1 in HCC cells. (A)** QRT-PCR analysis showing relative expression of miR-149 in HepG2 cells transfected with miR-149 mimics, miR-149 mimic control (negative control, NC), and blank control culture medium (mock). **(B, C and F)** Relative expression of AKT1, p-AKT1, mTOR and p-mTOR proteins in HepG2 cells transfected with miR-149 mimics, miR-149 mimic control (negative control, NC), and blank control culture medium (mock) detected by Western blot analysis. GAPDH was used as an internal loading control. **(D)** RNA sequence alignment showing the 3′-UTR of AKT1 mRNA contains a complementary site for the seed region of miR-149. AKT1mut is amutant with substitutions in the complementary region as a negative control. **(E)** Luciferase report assay was performed to verify whether AKT1 was a direct target of miR-149. The luciferase activity was detected after transfection of FLuci vector (3′-UTR-AKT1wt FLuci vector or 3′-UTR-AKT1mut FLuci vector) into the miR-149 mimic or miR-149 mimic control (negative control, NC) transfected HepG2 cells.

To verify the regulatory effect of miR-149 on AKT1 in HCC cells, we transfected HepG2 cells with miR-149 mimics, miR-149 mimic control (negative control, NC), and blank control culture medium (mock). After 24 h post-transfection, the expression level of AKT1 protein in HepG2 cells overexpressed miR-149 was significantly lower than those in NC and mock groups (both P < 0.001, Figure 3A ~ C).To verify whether AKT1 was a direct target of miR-149, the luciferase reporter containing the complimentary seed sequence of miR-149 at the 3′-UTR region of AKT1 mRNA was constructed (Figure 3D). Luciferase activity was detected at 48 h after the co-transfection of FLuci vector (3′-UTR-AKT1wt FLuci vector or 3′-UTR-AKT1mut FLuci vector), miR-149 mimic or NC mimic, and RLuci vector in HepG2 cells. As shown in Figure 3E, the luciferase activity was significantly decreased in HepG2 cells co-transfected with 3′-UTR-AKT1wt FLuci vector and miR-149 mimic compared with those co-transfected with 3′-UTR-AKT1mut FLuci vector and miR-149 mimic (P = 0.006), suggesting that the fragment at the 3′-UTR of the AKT1 mRNA was the complementary site for the miRNA-149 seed region, and thus, that AKT1 was a direct target of miR-149.

### MiR-149 regulates the AKT1/mTOR pathway in HCC cells

To verify the regulatory effect of miR-149 in AKT/mTOR pathway, we examined the expression of key components in this pathway, including AKT1, pAKT1, mTOR and pmTOR, in HepG2 cells with or without miR-149 overexpression. As shown in Figure 3B and F, the total and phosphorylated protein expression levels of AKT1 and mTOR were all significantly decreased in HepG2 cells overexpressed miR-149 (all P < 0.001), indicating that miR-149 might be an important regulator of this signaling pathway.

### Reverse correlation between miR-149 and AKT1 mRNA expression in human HCC tissues and cells

**Figure 4:**
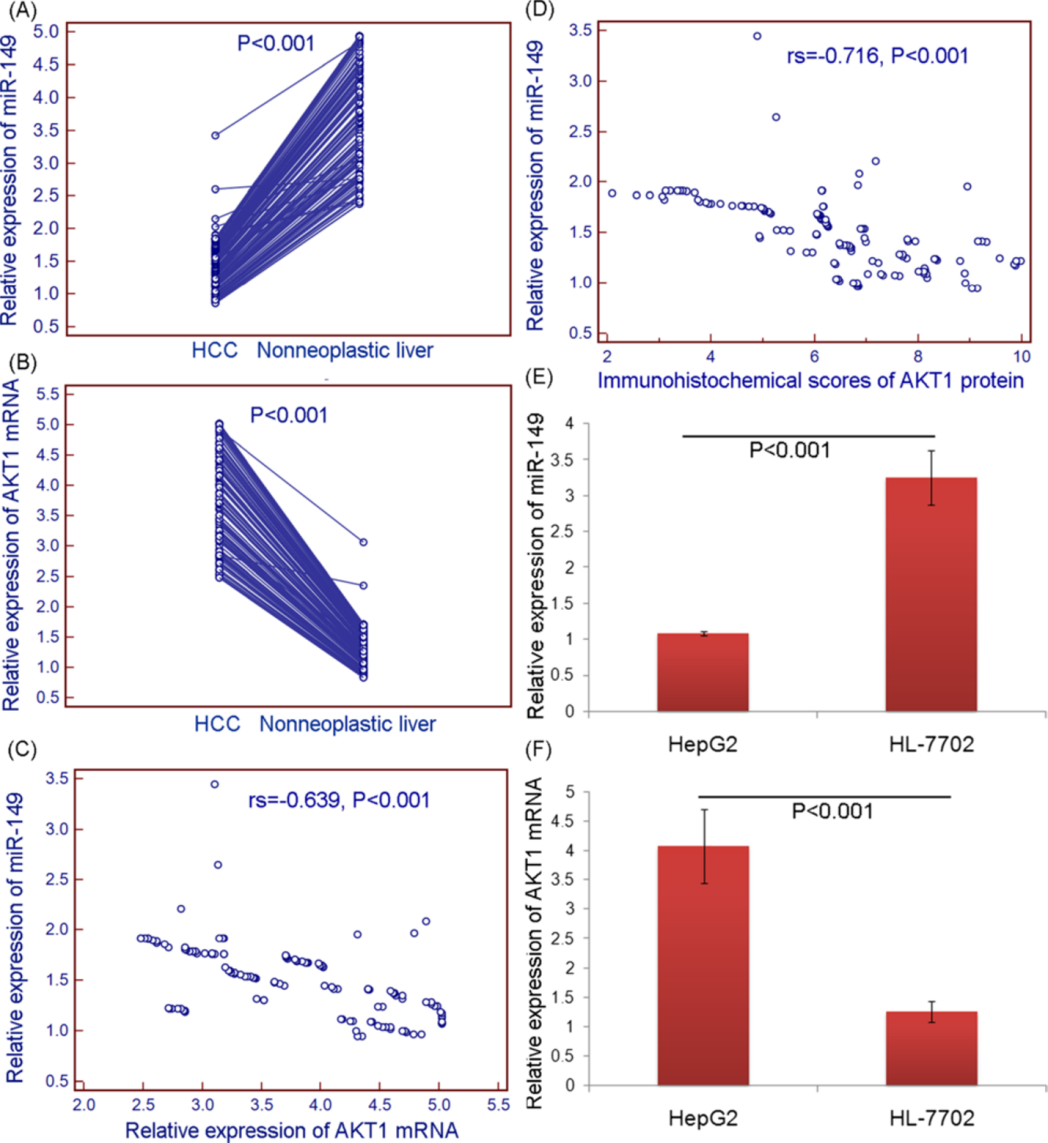
**Reverse correlation between miR-149 and AKT1 mRNA expression in human HCC tissues and cells. (A and B)** Relative expression of miR-149 and AKT1 mRNA in 130 self-pairs of HCC and adjacent nonneoplastic liver tissues; **(C)** Spearman Correlation analysis clearly showed a negative correlation between miR-149 and AKT1 mRNA expression in HCC tissues (rs = -0.639, P < 0.001); **(D)** Spearman Correlation analysis clearly showed a negative correlation between miR-149 and AKT1 protein expression in HCC tissues (rs = -0.716, P < 0.001); **(E and F)** Relative expression of miR-149 and AKT1 mRNA HCC (HepG2) and normal liver (HL-7702) cells.

QRT-PCR was performed to evaluate the relationship between miR-149 and AKT1 mRNA expression in HCC tissues and cells *in vitro*. As shown in Figure 4A and B, the expression level of miR-149 in HCC tissues was significantly lower than that in adjacent nonneoplastic liver tissues (P < 0.001, Figure 4A), while AKT1 mRNA expression was dramatically increased in HCC tissues compared to adjacent nonneoplastic liver tissues (P < 0.001, Figure 4B), which was similar to our previous study on the expression levels of AKT1 protein by immunohistochemistry (IHC) analysis based on the same cohort of HCC patients (IHC scores of AKT1 protein in HCC tissues vs. adjacent nonneoplastic liver tissues, mean ± S.D.: 6.32 ± 1.74 vs. 2.48 ± 0.29, P < 0.001) [15]. More interestingly, the Spearman Correlation analysis clearly showed negative correlations between miR-149 and AKT1 mRNA (protein, IHC scores) expression in HCC tissues (for miR-149 and AKT1 mRNA: rs = -0.639, P < 0.001, Figure 4C; for miR-149 and AKT1 protein: rs = -0.716, P < 0.001, Figure 4D). These findings were consistent with those based on HCC cells in vitro system (Figure 4E ~ F).

### MiR-149 inhibits cell proliferation, invasion and migration of HCC cells in vitro by targeting AKT1/mTOR pathway

**Figure 5:**
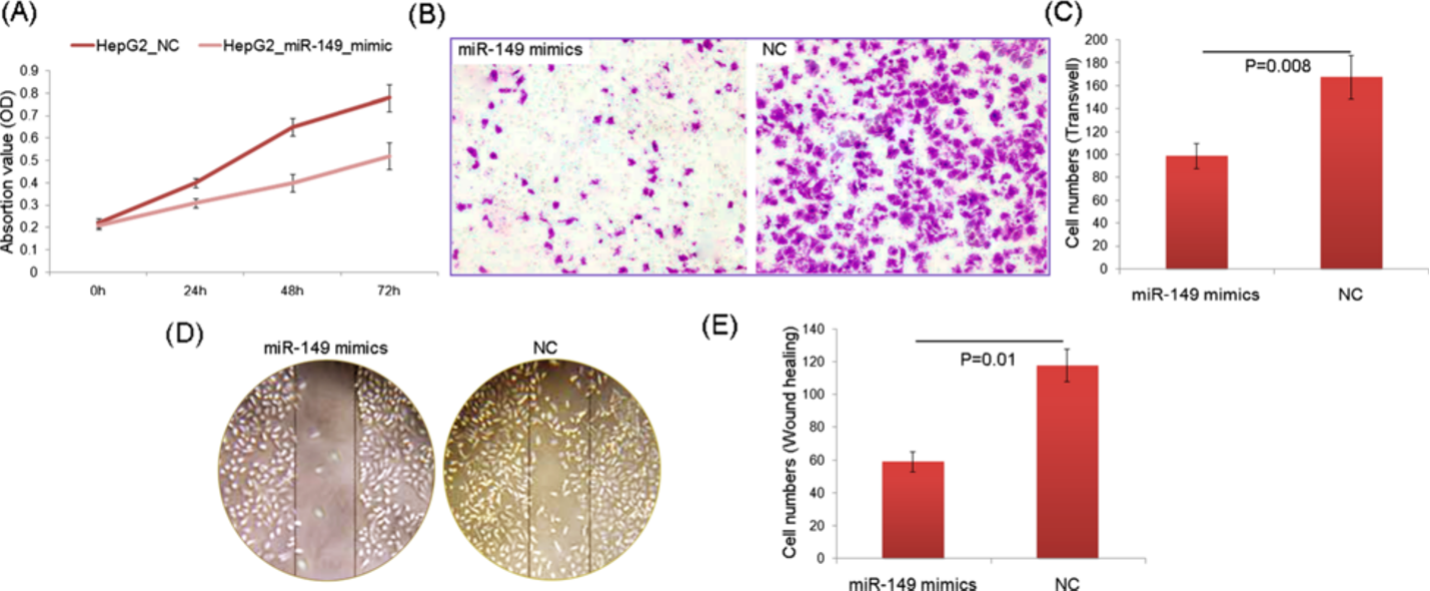
**MiR-149 inhibits cell proliferation, invasion and migration of HCC cells in vitro. (A)** MTT assay showed that miR-149 overexpression could inhibit cell proliferation of HepG2 cells. **(B and C)** Transwell analysis showed miR-149 overexpression could inhibit invasion of HepG2 cells. **(D and E)** Scratch assays showed miR-149 overexpression could inhibit migration of HepG2 cells.

**Figure 6:**
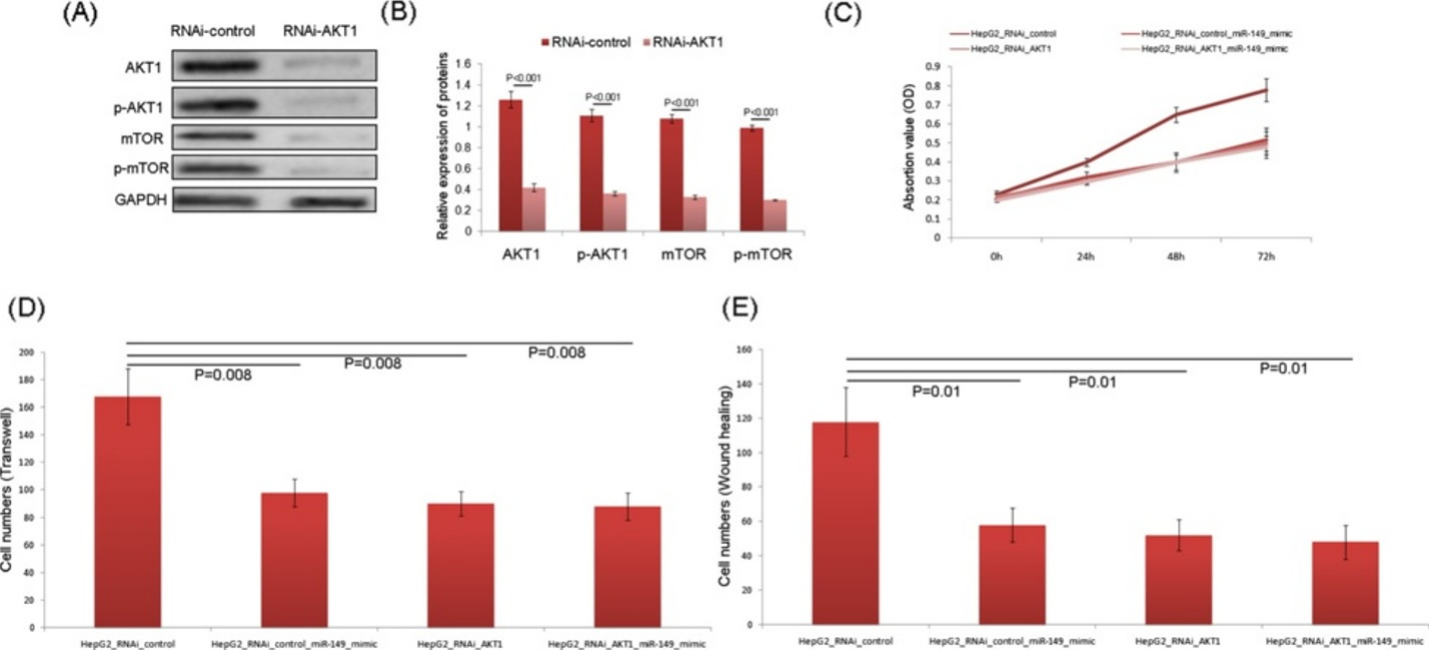
**MiR-149 inhibits cell proliferation, invasion and migration of HCC cells in vitro by targeting AKT1/mTOR pathway. (A and B)** Western blot analysis of AKT1, p-AKT1, mTOR and mTOR protein expression levels in HepG2 cells transfected with RNAi-AKT1 or RNAi-control vectors. **(C-E)** Overexpression of miR-149 fails to inhibit proliferation, invasion, and migration in AKT1 knockdown HepG2 cells.

The down-regulation of miR-149 in HCC cells and tissues prompted us to verify whether it acted as a tumor suppressor in this malignancy. As shown in Figure 5A, the enforced expression of miR-149 significantly inhibited cell proliferation of HepG2 cells (P = 0.01). In addition, transwell assays revealed that overexpression of miR-149 could dramatically reduce the invasion activities of HepG2 cells compared with those of control cells (P = 0.008, Figure 5B and C). Moreover, wound-healing assays also demonstrated that overexpression of miR-149 could markedly reduce the migration activities of HepG2 cells (P = 0.01, Figure 5D and E).To further verify whether the role of miR-149 in tumorigenesis of HCC was mediated via targeting AKT1-mTOR signaling, AKT1 expression in HepG2 cells was knockdown via siRNA, which resulted in the downregulation of AKT1, p-AKT1, mTOR and mTOR proteins (Figure 6A and B). Furthermore, knockdown of AKT1 expression abolished effects of miR-149 overexpression in HepG2 cells, including inhibiting cell proliferation (Figure 6C), invasion (Figure 6D) and migration (Figure 6E), indicating that the tumor suppressive roles of miR-149 in HCC were partially mediated by targeting AKT1-mTOR pathway.

### Down-regulation of miR-149 associates the advanced tumor progression and poor prognosis of human HCC

To evaluate whether miR-149 expression was associated with clinicopathological features of patients with HCC, we analyzed the association of miR-149 expression with T stage, tumor grade, presence of cirrhosis, underlying liver disease including alcohol abuse, viral hepatitis B and C, sex, and age (Table 1). In order to classify all 130 HCC patients into high miR-149 expression and low miR-149 expression groups, a cutoff value for miR-149 expression levels was chosen on the basis of a measure of heterogeneity with the log-rank test statistic with respect to overall survival. As a result, there were 70 (70/130, 53.85) patients belonging to low miR-149 expression group and 60 (60/130, 46.15%) patients belonging to low miR-149 expression group. Statistical analysis showed that the down-regulation of miR-149 was more frequently found in HCC tissues with high tumor stage (T3 ~ 4) than those with low tumor stage (T1 ~ 2, P = 0.01, Table 1).

**Table 1:** **Association of miR-149 expression with clinicopathologic features of 130 hepatocellular carcinoma patients**

Clinicopathologic features	Case	miR-149-low (n, %)	P
**Age (years)**			
≤50	72	38 (52.78)	NS
>50	58	32 (55.17)	
**Gender**			
Male	96	50 (52.08)	NS
Female	34	20 (58.82)	
**Serum AFP**			
Positive	72	42 (58.33)	NS
Negative	58	28 (48.28)	
**Tumor stage**			
T1	23	6 (26.09)	0.01
T2	40	15 (37.50)	
T3	52	34 (65.38)	
T4	15	15 (100.00)	
**Tumor grade**			
G1	31	16 (51.61)	NS
G2	76	39 (51.32)	
G3	23	15 (65.22)	
**Growth pattern**			
Trabecular	101	53 (52.48)	NS
Nontrabecular	29	17 (58.62)	
**Cirrhosis**			
Yes	86	45 (52.33)	NS
No	44	25 (56.82)	
**Underlying liver disease**			
Alcoholic	25	13 (52.00)	NS
Hepatitis B	49	28 (57.14)	
Hepatitis C	35	17 (48.57)	
Unknown	21	12 (57.14)	

Note: ‘NS’ refers to the differences among groups have no statistical significance.

**Figure 7:**
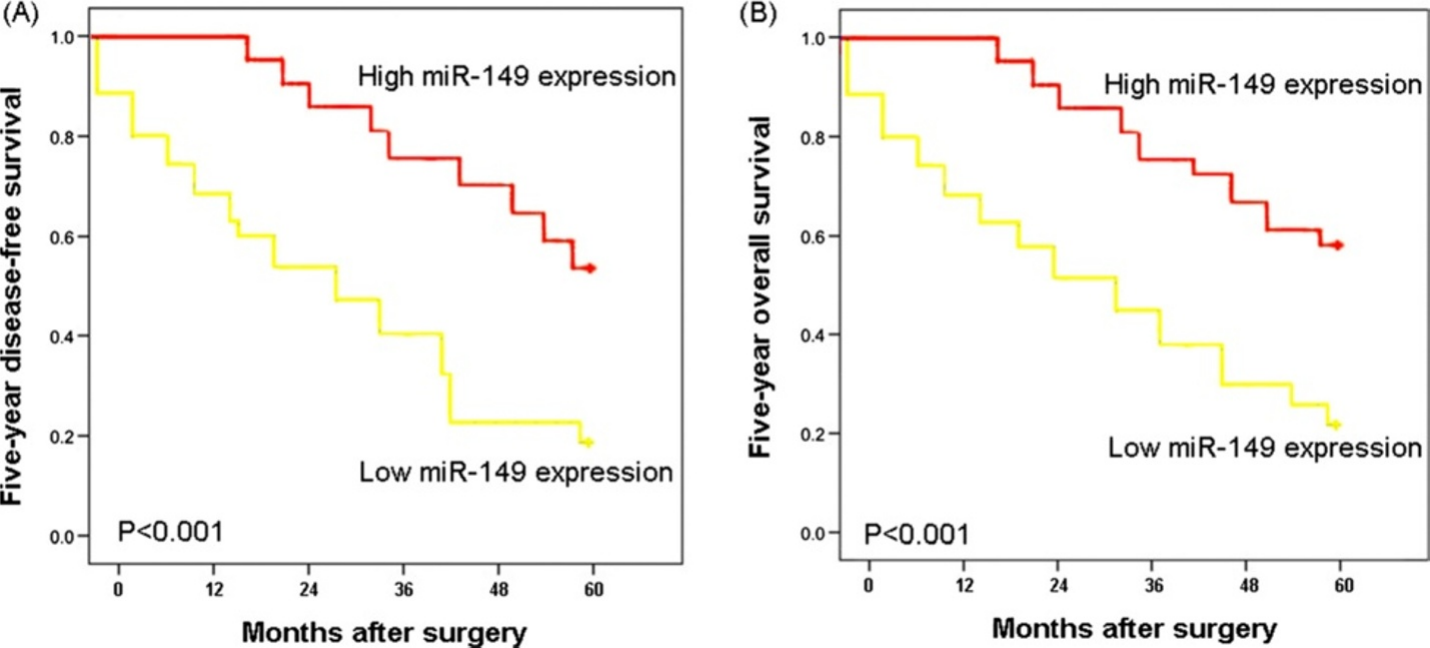
**Disease-free survival (A) and overall survival (B) curves for two groups defined by low and high expression of miR-149 in patients with HCC.** The patients with low miR-149 expression had a significantly shorter 5-year overall and disease-free survival rate than those with high expression (all P < 0.001).

To determine the prognostic value of miR-149 in patients with HCC, the Kaplan–Meier method was employed to analyze the correlation between miR-149 expression with 5-year disease-free survival and 5-year overall survival of HCC patients. As shown in Figure 7A, we observed a trend that 5-year disease-free survival of HCC patients with low miR-149 expression was significantly poorer than those with high miR-149 expression (P < 0.001, log-rank test; Figure 7A). Similarly, the Kaplan-Meier plot of 5-year overall survival curves stratified by miR-149 expression also showed a significant relationship between miR-149 expression and 5-year overall survival (P < 0.001, log-rank test, Figure 7B). Furthermore, the multivariate analysis found that miR-149 expression was an independent poor prognostic factor for both 5-year disease-free survival (P = 0.003, Table 2) and 5-year overall survival (P = 0.006, Table 2) in HCC.

**Table 2:** **Multivariate survival analysis of five-year overall and disease-free survival in 130 patients with hepatocellular carcinoma**

Features	Five-year overall survival			Five-year disease-free survival		
	HR	95% CI	P	HR	95% CI	P
Age	1.132	0.316-3.516	0.192	1.536	0.322-3.736	0.125
Gender	1.191	0.345-3.857	0.136	1.559	0.357-3.831	0.131
Serum AFP	1.931	0.685-4.056	0.063	1.953	0.615-4.273	0.062
Tumor stage	2.879	1.366-5.196	0.009	2.686	1.386-6.009	0.01
Tumor grade	1.563	0.609-4.088	0.081	1.551	0.607-4.466	0.086
Presence of cirrhosis	1.919	0.738-4.102	0.063	1.921	0.793-4.219	0.062
miR-149 expression	3.938	1.472-8.038	0.003	3.608	1.431-7.686	0.006

## Discussion

Elucidating the molecular mechanisms of tumor progression and tumor prognosis in human HCC remains largely unexplored. In the current study, we identified 6 candidate miRNAs targeting AKTs using an improved prediction protocol with two steps: (1), 5 different programs for miRNA target prediction were used due to their various algorithms and we identified the overlap which was the consensus among 3 out of 5 different programs; (2), we selected miRNA-target interactions (MTIs) with low hybridization energies. These two steps were performed to minimize false positive and false negative results. According to the enrichment analysis on GO items and KEGG pathways, we found that the most common functions of the experimentally validated targets of candidate miRNAs targeting AKTs included focal adhesion, cell cycle, p53 signaling pathway, mTOR signaling pathway, apoptosis, VEGF signaling pathway, which cover all the hallmarks of HCC, providing a convincing evidence that these candidate miRNAs may have a definitive impact on hepatocarcinogenesis. In order to improve our understanding of this candidate miRNAs on HCC malignant progression, we constructed the protein interaction network of their validate targets. Following the network topological analysis, we found that AKT1 and its interaction with mTOR respectively had the highest node-betweenness and edge-betweenness. Many previous studies have recognized highly connected nodes (hubs) as the most important points in the network. Very recently, growing reports have put forward a complementary notion that is bottlenecks as nodes/edges with a high betweenness centrality (i.e., network nodes/edges which have many ‘shortest paths’ going through them). In fact, bottlenecks represent key connector proteins/interactions with surprising functional and dynamic properties [20]. On this context, AKT1-mTOR may be the most important interaction in our miRNAs-regulated protein interaction network. Thus, we further performed a systematic experiments to validate the regulatory effect of a candidate miRNA targeting AKT1-miR-149 on AKT1-mTOR axis, and its involvement in hepatocarcinogenesis and clinical progression of HCC.

MiR-149, located on chromosome 2 [21], has been reported to play controversial roles in the progression of various types of human cancers. It is downregulated in several cancer cells and functions as a tumor suppressor by targeting oncogenes. For example, Chan et al. [22] reported that miR-149 could suppress breast cancer cell migration/invasion and metastasis by targeting GIT1; Cheng et al. [23] observed the decreased expression of miR-149 in clear cell renal cell carcinoma; Ke et al. [24] found that the expression of miR-149 was downregulated in lung cancer and miR-149 could inhibit EMT by targeting FOXM1; Pan et al. [18] indicated that miR-149 may play a tumor suppressive role in the proliferation and invasion of glioma cells via blockade of the AKT1 signaling. In contrast, miR-149 is also upregulated in several cancer cells and functions as a oncomiRs by targeting certain tumor suppressive genes. For example, miR-149 was highly expressed in nasopharyngeal carcinoma and promoted malignant progression by suppressing the expression of its target gene, Smad2 [25]; Its oncogenic role was also demonstrated in melanoma in which miR-149 upregulation caused the downregulation of GSK3- and upregulation of Mcl-1, leading to apoptotic resistance [26]. According to miRTarBase (Release 4.5: Nov. 1, 2013; http://mirtarbase.mbc.nctu.edu.tw/), E2F1 and MYBL2, together with AKT1, are validated targets for miR-149. Chen and colleagues reported that the upregulation of E2F1 protein might associate with worse outcomes in patients with HCC [27]. Frau and colleagues also indicated that MYBL2 upregulation could induce fast growth and progression of premalignant and malignant liver, through cell cycle deregulation and activation of genes and pathways related to tumor progression [28]. Nakajima and colleagues identified MYBL2 as a probable transcriptional target of E2F1 in HCC and as a useful biomarker for diagnosis and an attractive target for molecular therapies useful to treat HCC [29]. In the present study, our data showed the downregulated expression of miR-149 in HCC cell line and clinical specimens compared with the normal liver cell and matched adjacent nonneoplastic liver tissues, respectively. In addition, our functional study showed that AKT1 was a downstream target and effector of miR-149. Enforced expression of miR-149 and knockdown of AKT1 both inhibited the cell proliferation and tumorigenicity of HCC cell line in vitro. Strikingly, miR-149 overexpression did not inhibit the cell proliferation and tumorigenicity of HCC cells in vitro when AKT1 was knocked down. Then, our investigation of the downstream effectors of miR-149-AKT1 signaling axis showed that enforced expression of miR-149 and knockdown of AKT1 both could impair the activation of AKT1 and mTOR. Our data here provide the first and direct evidence that miR-149 has an tumor suppressive role in HCC cells by regulating AKT1/mTOR signaling, which was similar with the previous findings of Pan et al. [18] on glioma cells. More importantly, our data showed that the expression level of miR-149 was associated with tumor stage of HCC patients. Advance tumor stage samples have lower miR-149 expression compared to early tumor stage samples. These findings were similar to the data of Wang et al. on gastric cancer [30]. Furthermore, we also identified miR-149 as an independent prognostic factor for both 5-year disease-free survival and 5-year overall survival of HCC patients.

## Conclusion

This comprehensive analysis identified a list of miRNAs targeting AKTs and revealed their critical roles in HCC malignant progression. Especially, miR-149 may function as a tumor suppressive miRNA and play an important role in inhibiting the HCC tumorigenesis by modulating the AKT/mTOR pathway. Our clinical evidence also highlight the prognostic potential of miR-149 in HCC. The newly identified miR-149/AKT/mTOR axis might be a promising therapeutic target in the prevention and treatment of HCC.

## Materials and methods

### Prediction of miRNAs targeting AKTs

Candidate miRNAs which target AKT1, AKT2 and AKT3 were predicted by five different online programs, including TargetScan (Release 6.2, http://www.targetscan.org/) [31], miRDB (Last modified: April 03, 2012. http://mirdb.org/miRDB/) [32], DIANA-microT (DIANA-microT-CDS v5.0, http://diana.imis.athena-innovation.gr/ DianaTools/index.php?r = microT_CDS/index) [33], miRanda (Last Update: 2010-11-01, http://www.microrna.org/microrna/home.do) [34] and miRWalk (Last Update: 15th March 2011. http://www.umm.uni-heidelberg.de/apps/zmf/mirwalk/) [35], which represent different algorithms based on diverse features, thus, their combination could ensure high specificity of the prediction. miRNAs which were commonly predicted by more than 3 out of 5 programs were retained.

Then, RNAhybrid [36] was used to calculate the MFE of the duplex miRNA:mRNA. We chose miRNA-AKTs interactions, MFE values of which were lower than the median of all MFE values, as candidate miRNA-AKTs interaction.

After that, validated targets for above candidate miRNAs which target AKTs were collected from miRTarBase (Release 4.5: Nov. 1, 2013; http://mirtarbase.mbc.nctu.edu.tw/), which has accumulated more than fifty thousand MTIs [37]. Generally, the collected MTIs are validated experimentally by reporter assay, western blot, microarray and next-generation sequencing experiments. Here, we only collected the MTIs which are validated experimentally by reporter assay, western blot and qPCR. In order to facilitate data analysis, the different ID types for validated target genes were converted to ID from UniProtKB-Swiss-Prot (release-2014_02).

### GO and pathway enrichment analysis for validated genes of candidate miRNAs which target AKTs

We used Database for Annotation, Visualization and Integrated Discovery [38] (DAVID, http://david.abcc.ncifcrf.gov/home.jsp,version%206.7) for GO enrichment analysis. We also performed pathway enrichment analysis using pathway data obtained from the FTP service of KEGG [39] (Kyoto Encyclopedia of Genes and Genomes, http://www.genome.jp/kegg/, Last updated: Oct 16, 2012).

### Network analysis

#### Protein-protein interaction (PPI) data

PPI data were imported from eight existing PPI databases including Human Annotated and Predicted Protein Interaction Database (HAPPI) [40], Reactome [41], Online Predicted Human Interaction Database (OPHID) [42], InAct [43], Human Protein Reference Database (HPRD) [44], Molecular interaction Database (MINT) [45], Database of Interacting Proteins (DIP) [46], and PDZBase [47]. The detailed information on these PPI databases is described in Additional file 9: Table S8.

### Network construction

Interactions between proteins encoded by validated target genes of candidate miRNAs which target AKTs were used to construct miRNA-regulated protein interaction networks. The PPI data were obtained from eight existing PPI databases as mentioned above. Then, we applied Navigator software (Version 2.2.1) and Cytoscape (Version 2.8.1) to visualize the networks.

### Defining network topological feature set

For each node i in miRNA-regulated protein interaction network, we defined four measures for assessing its topological property: (1) ‘Degree’ is defined as the number of links to node i; (2) ‘Node betweenness’ is defined as the number of shortest paths between pairs of nodes that run through node i. (3) ‘Closeness’ is defined as the inverse of the farness which is the sum of node i distances to all other nodes. The Closeness centrality can be regarded as a measure of how long it will take to spread information from node i to all other nodes sequentially. Degree, betweenness and closeness centralities can measure a protein’s topological importance in the network. The larger a protein’s degree/node betweenness/closeness centrality is, the more important the protein is in the PPI network [48]. (4) ‘Modularity’: proteins that are highly interconnected within the network are usually involved in the same biological modules or pathways. Here, we used a Markov clustering algorithm to divide all nodes into different functional modules.

For each edge in miRNA-regulated protein interaction network, we calculated its ‘Edge Betweenness’ to assess the importance of a specific interaction in the network. ‘Edge Betweenness’ is defined as the frequency of an edge that places on the shortest paths between all pairs of vertices in network [49]. The edges with highest betweenness values are most likely to lie between functional modules.

### Experimental validation

#### Patients and Tissue Samples

The study was approved by the Research Ethics Committee of 302 Hospital of PLA, Beijing, China. Written informed consent was obtained from all patients. All specimens were handled and made anonymous according to the ethical and legal standards.

A total of 130 self-pairs of HCC specimens and adjacent nonneoplastic liver tissues were snap-frozen in liquid nitrogen and stored at -80°C following surgery for qRT-PCR assay. All the tissues were obtained from 130 patients with primary HCC who underwent a curative liver resection at the 302nd Hospital of PLA, Beijing, China. These patients were diagnosed as HCC between 2001 and 2006. None of the patients recruited in this study had chemotherapy or radiotherapy before the surgery. HCC diagnosis was based on World Health Organization (WHO) criteria. Tumor differentiation was defined according to the Edmondson grading system. Liver function was assessed using the Child-Pugh scoring system. Tumor staging was determined according to the sixth edition of the tumor-node-metastasis (TNM) classification of the International Union against Cancer. The clinicopathological features of 130 patients are summarized in Table 1.

The median follow-up period was 8.6 years. Postoperative surveillance included routine clinical and laboratory examinations every third month, computed tomography scans of the abdomen, and radiographs of the chest every third month. After 5 years, the examination interval was extended to 12 months.

### Cell culture

Human HCC cell line HepG2 was obtained from the American Type Culture Collection (Manassas, VA, USA) and was cultured in DMEM (Invitrogen, USA) supplemented with 10% fetal bovine serum (Gibico, USA), 2 mM L-glutamine and antibiotics. Normal human liver cell line HL-7702 was obtained from the American Type Culture Collection (Manassas, VA, USA) and was maintained in RPMI 1640 medium (Invitrogen, USA) supplemented with 10% fetal bovine serum (Gibico, USA). Two cell lines were both maintained at 37°C in a humidified chamber supplemented with 5% CO_2_.

### Construction of miR-149 expression vectors and cellular transfection

Commercial miR-149 mimics and miR-149 mimic control expression vectors were purchased from Invitrogen Life Technologies, USA. The sequence information of two vectors were as following: for miR-149 mimic, 5′-UCU GGC UCC GUG UCU UCA CUC CC-3′, for miR-149 mimic control, 5′-GGG AGU GAA GAC ACG GAG CCA GA-3′. HepG2 cells were transfected with miR-149 mimics and miR-149 mimic control expression vectors with Lipofectamine 2000 reagent (Invitrogen Life Technologies, USA) according to the manufacturer’s protocol.

### QRT-PCR

The qRT-PCR analysis for miRNA and mRNA was performed according to the similar protocol of our previous studies [13, 50]. U6 small RNA and GAPDH were respectively used as internal controls for normalization and quantification miR-149 and AKT1 expression. The primer sequences were listed in Additional file 10: Table S9. Relative quantification of miRNA and mRNA expression was evaluated using the comparative cycle threshold (CT) method. All experiments were done in triplicate. Mean normalized gene expression ± SE was calculated from independent experiments.

### RNA interference and cellular transfection

To knockdown the expression of the human Akt1 gene, pGCSIL-GFP-Akt1 small hairpin RNA (NM_005163), an Akt1-RNA interference (RNAi) lentiviral vector (RNAi-AKT1), was constructed (Shanghai GeneChem Co, Ltd., Shanghai, China). The sense primer was 5′-CCGGGAGGCCAAGTCCTTGCTTTCAttcaagagaTGAAAGCAAGGACTTGGC- CTCTTTTTG-3′, and the anti-sense primer was 5′ -AATTCAAAAAGAGGCCAAGT-CCTTGCTTTCAtctcttgaaTGAAAGCAAGGACTTGGCCTC- 3. A scrambled short-hairpin RNA was used as a negative control (RNAi-control). HepG2 cells were seeded into 6-well plates and incubated overnight, and then transfected using LipofectamineTM 2000 transfection reagent (Invitrogen) according to the manufacturer’s instructions. The expression levels of AKT1 in HepG2 cells transfected with RNAi-AKT1 and RNAi-control were detected by Western blot analysis as described in the next section.

### Western blot

The Western blot protocol and semiquantitative analysis were carried out following the protocol of our previous studies [13, 50]. The specific antibodies were as following: AKT1 antibody (#sc-1618, goat polyclonal antibody, dilution 1:150, Santa Cruz Biotechnology, Inc. USA), p-AKT1 (Ser 473) antibody (#sc-7985, rabbit polyclonal antibody, dilution 1:150, Santa Cruz Biotechnology, Inc. USA), mTOR antibody (#sc-1549, goat polyclonal antibody, dilution 1:100, Santa Cruz Biotechnology, Inc. USA), p-mTOR (Ser 2448) antibody (#sc-101738, rabbit polyclonal antibody, dilution 1:100, Santa Cruz Biotechnology, Inc. USA), and GAPDH antibody (CW0266, dilution 1:1,000, CoWin Biotech). All experiments were done in triplicate. Mean normalized gene expression ± SE was calculated from independent experiments.

### Luciferase reporter assay

The regulatory effect of miR-149 to AKT1 was evaluated by a luciferase reporter assay in HepG2 cells following the protocol of our previous studies [13, 50]. Briefly, the human AKT1 3′-UTR luciferase reporter construct was generated by cloning AKT1 3′-UTR sequence containing the predicted miR-149 binding site into the 3′-UTR region of the pGL3 luciferase reporter vector (Promega Corporation, Madison WI, USA). The miR-149 binding site-deleted AKT1 3′-UTR luciferase reporter construct was generated by PCR fragments of AKT1 3′-UTR luciferase reporter construct lacking the target site and ligated. HepG2 cells were cultivated in 24-well plates and co-transfected using Fugene (Roche) with 100 ng of pGL3-AKT1-miR-149 constructs, 10 ng miR-149 mimic or NC mimic, and 2 ng pRL-SV40 RLuci vector (Promega). The luciferase activity assay was performed 24 h after transfection using the dual-luciferase reporter assay system (Promega Corporation, Madison WI) according to the manufacturer’s instructions.

### In vitro cell proliferation assay

The in vitro cell proliferation of HepG2 cells transfected with miR-149 mimic, miR-149 mimic control, RNAi-AKT1 and RNAi-control vectors, respectively, were measured using the 3-(4,5-dimethylthiazol-2- yl)-2,5-diphenyltetrazolium bromide (MTT) method following the protocol of our previous studies [13, 50]. All experiments were done in triplicate. Mean normalized gene expression ± SE was calculated from independent experiments.

### In vitro invasion and migration assay

Cell invasion and migration were respectively analyzed by Matrigel coated transwell cell culture chambers (8 μm pore size, Millipore, Billerica, MA, USA) and the scratch wound-healing motility assay following the protocol of our previous studies [13, 50]. All experiments were done in triplicate. Mean normalized gene expression ± SE was calculated from independent experiments.

### Statistical analysis

The software of SPSS version13.0 for Windows (SPSS Inc, IL, USA) and SAS 9.1 (SAS Institute, Cary, NC) was used for statistical analysis. The chi-squared test was used to show differences in categorical variables. Patient survival and the differences in patient survival were determined by the Kaplan-Meier method and the log-rank test, respectively. A Cox regression analysis (proportional hazard model) was performed for the multivariate analyses of prognostic factors. Differences were considered statistically significant when P was less than 0.05.

## Electronic supplementary material

Additional file 1: Table S1: MicroRNAs (miRNAs) which respectively regulate AKT1, AKT2 and AKT3 predicted by more than three miRNA target prediction programs. (DOC 46 KB)

Additional file 2: Table S2: Minimum free energy (MFE) of the duplex miRNA:AKTs calculated by RNAhybrid. (DOC 34 KB)

Additional file 3: Table S3: Validated targets for six candidate miRNAs collected from miRTarBase. (XLS 26 KB)

Additional file 4: Figure S1: Enriched gene ontology (GO) biological processes (A) and KEGG pathways (B) involved by involved by validated target genes of hsa-miR-149, hsa-miR-302d, hsa-miR-184, hsa-miR-708, hsa-miR-122 and hsa-miR-124. ‘*’ P < 0.01; ‘**’ P < 0.001. (TIFF 472 KB)

Additional file 5: Table S4: Interaction information of proteins encoded by validated targets of 6 candidate miRNAs. (XLS 279 KB)

Additional file 6: Table S5: Interaction information of hub nodes in the candidate miRNAs-regulated protein interaction network. (XLS 35 KB)

Additional file 7: Table S6: Topological features of 28 important nodes screened from miRNAs-regulated protein interaction network. (DOC 44 KB)

Additional file 8: Table S7: Edge betweenness for each interaction in the interaction network of hub proteins. (XLS 94 KB)

Additional file 9: Table S8: Detailed information on eight existing protein-protein interaction databases. (XLS 20 KB)

Additional file 10: Table S9: MicroRNAs (miRNAs) and mRNAs detected by qRT-PCR and their primers. (DOC 29 KB)
