# Supplementary material for: Comprehensive analysis of microRNA-regulated protein interaction network reveals the tumor suppressive role of microRNA-149 in human hepatocellular carcinoma via targeting AKT-mTOR pathway
Source: Mol Cancer. 2014 Nov 26;13:253. doi: 10.1186/1476-4598-13-253 (PMC4255446; doi:10.1186/1476-4598-13-253)
Supplement: Supplementary file 1 — Additional file 1: Table S1: MicroRNAs (miRNAs) which respectively regulate AKT1, AKT2 and AKT3 predicted by more than three miRNA target prediction programs. (DOC 46 KB) [file 12943_2014_1452_MOESM1_ESM.doc]

**Table S1 MicroRNAs (miRNAs) which respectively regulate AKT1, AKT2 and AKT3 predicted by more than three miRNA target prediction programs**

| **AKT1** | | **AKT2** | | **AKT3** | |
| --- | --- | --- | --- | --- | --- |
| **MiRNA** | **Number of methods** | **MiRNA** | **Number of methods** | **MiRNA** | **Number of methods** |
| hsa-miR-149 | 4 | hsa-miR-137 | 5 | hsa-miR-124 | 4 |
| hsa-miR-302a | 3 | hsa-miR-184 | 4 | hsa-miR-15a | 4 |
| hsa-miR-302b | 3 | hsa-miR-29a | 3 | hsa-miR-16 | 4 |
| hsa-miR-302c | 3 | hsa-miR-29b | 3 | hsa-miR-17 | 4 |
| hsa-miR-302d | 3 | hsa-miR-29c | 3 | hsa-miR-195 | 4 |
|  |  | hsa-miR-708 | 3 | hsa-miR-320d | 4 |
|  |  |  |  | hsa-miR-424 | 4 |
|  |  |  |  | hsa-miR-497 | 4 |
|  |  |  |  | hsa-miR-122 | 3 |
|  |  |  |  | hsa-miR-181a | 3 |
|  |  |  |  | hsa-miR-181b | 3 |
|  |  |  |  | hsa-miR-181c | 3 |
|  |  |  |  | hsa-miR-181d | 3 |
|  |  |  |  | hsa-miR-320a | 3 |
|  |  |  |  | hsa-miR-320b | 3 |
|  |  |  |  | hsa-miR-320c | 3 |
|  |  |  |  | hsa-miR-376a | 3 |
|  |  |  |  | hsa-miR-532 | 3 |
