# Supplementary material for: Comprehensive analysis of microRNA-regulated protein interaction network reveals the tumor suppressive role of microRNA-149 in human hepatocellular carcinoma via targeting AKT-mTOR pathway
Source: Mol Cancer. 2014 Nov 26;13:253. doi: 10.1186/1476-4598-13-253 (PMC4255446; doi:10.1186/1476-4598-13-253)
Supplement: Supplementary file 2 — Additional file 2: Table S2: Minimum free energy (MFE) of the duplex miRNA:AKTs calculated by RNAhybrid. (DOC 34 KB) [file 12943_2014_1452_MOESM2_ESM.doc]

**Table S2 Minimum free energy (MFE) of the duplex miRNA:AKTs calculated by RNAhybrid**

| **Duplex miRNA:mRNA** | **MFE value (kcal/mol)** | **Sum of MFE (kcal/mol)** |
| --- | --- | --- |
| **hsa-miR-149:AKT1** | **-27.70/-24.40/-18.60** | **-70.50** |
| hsa-miR-302a:AKT1 | -13.50/-5.30/-9.30 | -28.10 |
| hsa-miR-302b:AKT1 | -12.30/-6.60/-10.02 | -28.92 |
| hsa-miR-302c:AKT1 | -14.50/-7.20/-8.80 | -30.50 |
| **hsa-miR-302d:AKT1** | **-15.60/-8.70/-9.70** | **-34.00** |
| hsa-miR-137:AKT2 | -10.00/-9.30/-11.90 | -31.20 |
| **hsa-miR-184:AKT2** | **-26.20/-17.60/-14.80** | **-58.60** |
| **hsa-miR-708:AKT2** | **-15.10/-17.70/-14.60** | **-47.40** |
| **hsa-miR-122:AKT3** | **-18.70/-9.70/-6.10** | **-34.50** |
| hsa-miR-16-5p:AKT3 | -12.10/-8.60/-7.30 | -28.00 |
| hsa-miR-15a-5p:AKT3 | -13.60/-10.10/-9.00 | -32.70 |
| **hsa-miR-124:AKT3** | **-10.80/-10.30/-13.80** | **-34.90** |

**Note:** Data with bold marks refer to 6 optimized miRNAs-AKTs interactions with lower MFEs than the median values [-33.35(kcal/mol)] of all MFEs.
