# Supplementary figures and images for: Comprehensive analysis of microRNA-regulated protein interaction network reveals the tumor suppressive role of microRNA-149 in human hepatocellular carcinoma via targeting AKT-mTOR pathway
Source: Mol Cancer. 2014 Nov 26;13:253. doi: 10.1186/1476-4598-13-253 (PMC4255446; doi:10.1186/1476-4598-13-253)

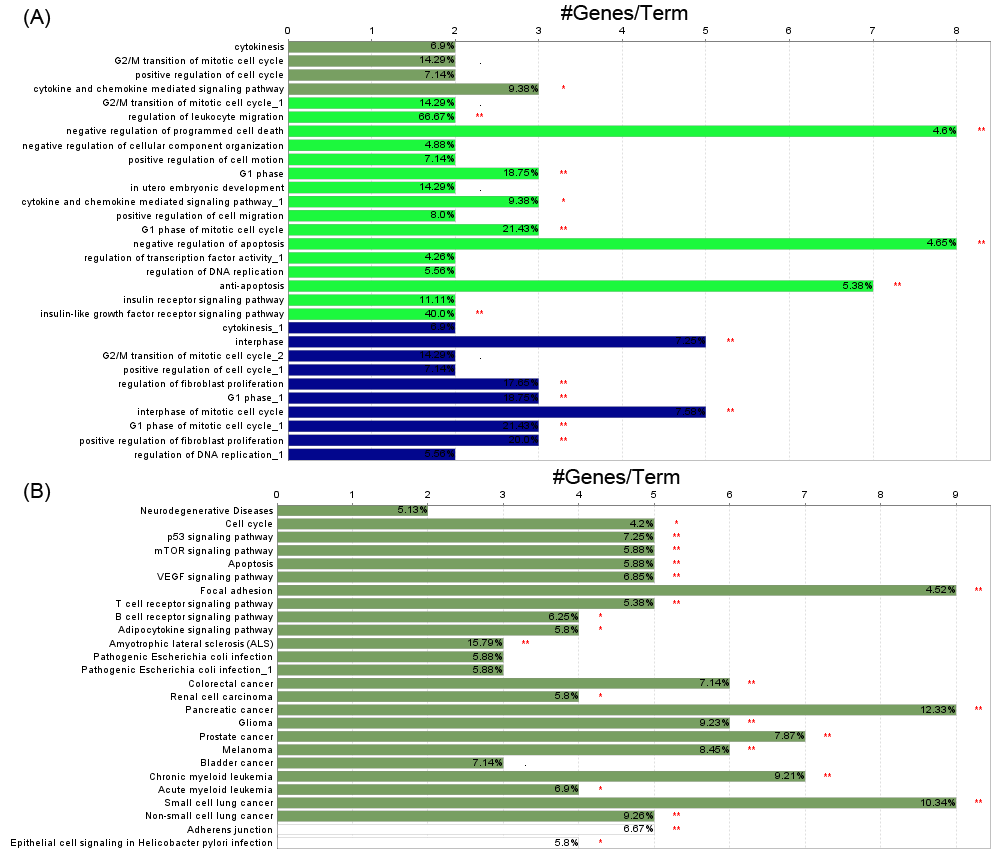

Supplement: Supplementary file 4 — Additional file 4: Figure S1: Enriched gene ontology (GO) biological processes (A) and KEGG pathways (B) involved by involved by validated target genes of hsa-miR-149, hsa-miR-302d, hsa-miR-184, hsa-miR-708, hsa-miR-122 and hsa-miR-124. ‘*’ P < 0.01; ‘**’ P < 0.001. (TIFF 472 KB) [file 12943_2014_1452_MOESM4_ESM.tiff]
