# Supplementary material for: Comprehensive analysis of microRNA-regulated protein interaction network reveals the tumor suppressive role of microRNA-149 in human hepatocellular carcinoma via targeting AKT-mTOR pathway
Source: Mol Cancer. 2014 Nov 26;13:253. doi: 10.1186/1476-4598-13-253 (PMC4255446; doi:10.1186/1476-4598-13-253)
Supplement: Supplementary file 7 — Additional file 7: Table S6: Topological features of 28 important nodes screened from miRNAs-regulated protein interaction network. (DOC 44 KB) [file 12943_2014_1452_MOESM7_ESM.doc]

**Table S6 Topological features of 28 important nodes screened from miRNAs-regulated protein i**nteraction network

| **Topological important nodes** | **Degree** | **nCloseness** | **nBetweenness** |
| --- | --- | --- | --- |
| AKT1 | 32 | 54.44 | 18.91 |
| RELA | 21 | 50.00 | 11.48 |
| AR | 16 | 47.34 | 6.18 |
| RAC1 | 20 | 46.45 | 7.51 |
| AKT2 | 13 | 45.58 | 2.41 |
| AKT3 | 13 | 45.16 | 2.58 |
| STAT3 | 10 | 45.16 | 3.11 |
| MAPK3 | 11 | 44.95 | 4.99 |
| PTPN1 | 12 | 44.55 | 4.17 |
| NFKB1 | 10 | 44.14 | 2.11 |
| HSP90AA1 | 9 | 43.56 | 3.09 |
| E2F1 | 17 | 43.17 | 3.47 |
| CASP3 | 10 | 42.79 | 1.25 |
| MYBL2 | 11 | 42.42 | 3.70 |
| RHOA | 13 | 42.06 | 2.08 |
| SOCS1 | 10 | 41.88 | 2.95 |
| IGF1R | 8 | 41.53 | 1.22 |
| NR3C1 | 12 | 41.00 | 2.26 |
| CDK6 | 15 | 40.83 | 1.71 |
| JUN | 8 | 40.83 | 3.11 |
| MTOR | 10 | 46.83 | 9.56 |
| SP1 | 8 | 39.36 | 3.34 |
| CDK2 | 23 | 48.04 | 8.73 |
| CDK4 | 23 | 44.75 | 5.93 |
| MYC | 11 | 40.83 | 4.07 |
| BCL2 | 9 | 40.00 | 2.46 |
| CCND1 | 9 | 39.52 | 1.49 |
| GRB2 | 11 | 40.83 | 5.18 |
