# Supplementary material for: Comprehensive analysis of microRNA-regulated protein interaction network reveals the tumor suppressive role of microRNA-149 in human hepatocellular carcinoma via targeting AKT-mTOR pathway
Source: Mol Cancer. 2014 Nov 26;13:253. doi: 10.1186/1476-4598-13-253 (PMC4255446; doi:10.1186/1476-4598-13-253)
Supplement: Supplementary file 10 — Additional file 10: Table S9: MicroRNAs (miRNAs) and mRNAs detected by qRT-PCR and their primers. (DOC 29 KB) [file 12943_2014_1452_MOESM10_ESM.doc]

**Table S9 MicroRNAs (miRNAs) and mRNAs detected by qRT-PCR and their primers**

|  | **Primer sequences** |
| --- | --- |
| miR-149 | Forward: 5'- CAG TGC AGG GTC CGA GGT ATT -3'  Reverse: 5'- GGC TCT GGC TCC GTG TCT T -3' |
| RNU6 | Forward: 5'- AGT GCA GGG TCC GAG GTA TTC-3'  Reverse: 5'- CAA ATT CGT GAA GCG TTC CAT A-3' |
| AKT1 | Forward: 5'- GCT GGA CGA TAG CTT GGA-3'  Reverse: 5'- GAT GAC AGA TAG CTG GTG-3' |
| GAPDH | Forward: 5'-TGA TGA CAT CAA GAA GGT GGT GAA G-3'  Reverse:5'-TCC TTG GAG GCC ATG TGG GCC AT-3' |
